# An online-based intervention to promote healthy eating through self-regulation among children: study protocol for a randomized controlled trial

**DOI:** 10.1186/s13063-020-04685-5

**Published:** 2020-09-14

**Authors:** Paula Magalhães, Cátia Silva, Beatriz Pereira, Gabriela Figueiredo, Ana Guimarães, Armanda Pereira, Pedro Rosário

**Affiliations:** Department of Applied Psychology, School of Psychology, University of Minho, Campus de Gualtar, 4710-057 Braga, Portugal

**Keywords:** Self-regulation, eHealth, Healthy eating, Children, Protocol, Randomized controlled trial

## Abstract

**Background:**

Despite the enormous investment governments allocate to fight obesity, its worldwide prevalence is still on the rise. Moreover, the majority of the programs implemented are still targeting adults struggling with overweightness and focusing on transmitting knowledge about food. However, research shows that obesity prevention is more efficacious and cheaper, and beliefs about healthy eating have a stronger influence on eating behavior than declarative knowledge about food. In fact, knowledge about healthy eating only influences weight status when combined with self-regulation competences. Thus, the main goal of the current project is to develop and evaluate the efficacy of an online preventive intervention program, the HEP-S. This program is designed to promote and develop a set of transversal skills and strategies, related to self-regulation, on the healthy eating domain among school-aged children.

**Methods:**

A three-armed randomized controlled trial will be conducted in several schools in Portugal. It will include a standard control group, with no intervention; an online intervention group, with the program for 20 weeks; and an enhanced online intervention group, with the program for 20 weeks embedded with gamification strategies throughout the program. Per research group, 40 groups of about 15 children each will be recruited and measured at five different time points. The three research groups will complete the same assessment protocol at the same timings (baseline, post-intervention, and 3, 6, and 9 months’ follow-ups). The assessment protocol will include anthropometric and psychological measures. The primary outcome measures will be the development of self-regulation skills for healthy eating over time, the development of self-efficacy attitudes, knowledge about healthy eating over time, and others. The secondary outcome measures will include the effect of gamification strategies, engagement, and satisfaction with the program, among others. The program will comprise the following: (i) a weekly group synchronous videoconference session with a trained educational psychologist serving as a mediator and (ii) a weekly parental involvement activity. Narratives, or story-tools, embedded with self-regulation strategies are at the core of the intervention.

**Discussion:**

The program may play an important role in preventing risky and unhealthy eating behaviors by focusing on the development of self-regulation skills and strategies among elementary school children.

**Trial registration:**

ClinicalTrials.gov NCT04099498. Registered on 23 September 2019.

## Introduction

### Background

Obesity is a growing epidemic affecting individuals of all generations. According to the World Health Organization, obesity has more than tripled worldwide since 1980 [1]. The rate has doubled among children and tripled among adolescents globally [1–3], with a third of European youth now having overweightness or obesity [4]. Similarly, an increasing rate of childhood obesity has also been observed in the Portuguese population. A recent study from the National Food, Nutrition, and Physical Activity Survey [5] reported that 17.3% of children below the age of 10 were overweight and 7.7% were obese. Additionally, regarding adolescents (10 to 17 years old), 23.6% were overweight and 8.7% were classified as obese [5].

Child and adolescent obesity is not only associated with medical problems such as cardiovascular disease, hypertension, metabolic syndrome, and type 2 diabetes [1, 6], but also with social and psychological risks during childhood [7]. What is more, childhood obesity is a high predictor of obesity in adulthood [8, 9]. That is, youth with overweightness and obesity have an increased risk of maintaining their unhealthy weight status into adulthood [10]. The etiology of childhood overweightness and obesity is multifactorial and the result of a combination of genetic and environmental influences [11]. Still, most cases are associated with external and modifiable factors such as eating habits and physical inactivity [12–14].

The current state of affairs shows that despite the extensive amount of investment to tackle this worrying picture, through campaigns or specific programs [15], the rates of obesity do not seem to be reverting. A plethora of interventions that worked to prevent or reduce the overweightness and obesity have been implemented and evaluated (e.g., [15, 16]). Still, the results are elusive with small improvements at best. The majority of these interventions focused on transmitting nutritional knowledge about healthy food, especially emphasizing on increased consumption of fruits and vegetables, and targeting specific behaviors, such as unhealthy eating habits and lack of physical activity (e.g., [14]). However, research shows that beliefs about healthy eating have a stronger influence on eating behavior than factual knowledge about food [17] and that providing knowledge about healthy eating only has influence on weight when combined with the promotion of self-regulation competences [18]. This suggests that the traditional approach of simply conveying knowledge on healthy eating may not be the optimal one.

Moreover, interventions often lack a theoretical background underlying the activities [16]. The field of health is witnessing a shift from a disease model to a health promotion model [19, 20]. Consequently, there is a move from a “weight reduction” to a “healthy habits education” paradigm, i.e., promote self-control and prevent excessive food intake [21]. Additionally, there is increasing evidence that remedial approaches are less effective and costlier than preventive approaches [12]. Lastly, governments and relevant stakeholders are pressured by society at large that effective prevention and health promotion programs reach more individuals, particularly children.

Amidst this scenario, information and communication technologies (ICT) are emerging as innovative and potentially cost-effective ways to respond to these challenges. ICT seems to offer the possibility of lowering the costs associated with implementing intervention programs, with the added potential benefit of reaching more individuals. In fact, eHealth interventions are spreading in many domains, including healthy eating or weight management [22]. The results of online tools or web-based programs in promoting healthy lifestyles and behaviors (e.g., fruits and vegetables consumption) and in supporting obesity treatment are very encouraging, with real potential to create a sustained impact on prevention and treatment of obesity [22]. However, despite the increasing number of online interventions on this topic and its potential on behavioral change, there is still a need of further investigation. The results of the majority of these online interventions are unclear; the designs tend to be diverse and the duration of the interventions tends to be short; and, lastly, few interventions include weight loss strategies sustained on a theoretical framework [23]. Also, the actual reach of internet-delivered interventions seems to be lower, and attrition rates higher, than expected, which reinforces the need to further explore this mode of delivering the intervention. Lastly, there are few online interventions focusing on preventive obesity approach by promoting healthy eating strategies among the general population, especially in early stages of life (e.g., children), and not only for individuals at risk or struggling with obesity [24].

### Intervention strategy of HEP-S—the rationale

Healthy Eating Promotion through Self-regulation (HEP-S) program is grounded on the self-regulation (SR) approach, which has several features that make it unique when it comes to health promotion [19–21]. Grounded on Social Cognitive Theory, SR subsumes the processes that allow individuals to proactively control the personal, behavioral, and environmental influences that impact human behavior, including eating [25]. These processes are intrinsically cyclic and interdependent, open and dynamic, and proceed through three main phases: the preceding phase (forethought), the performance or volitional control phase, and the self-reflection phase [26] [or Planning, Execution, and Evaluation [25, 27]]. The SR process is under the influence of the individual and can, therefore, be taught and improved. Thus, the agent role of the individual throughout the process is key to become in control and autonomous.

Although individuals may be influenced and regulated by external factors and agents, exclusively relying on external regulation does not allow for the individual to develop adaptive competences and skills, such as choosing a healthy snack [20, 28]. Thus, to self-regulate their behavior and become increasingly autonomous, individuals must progressively shift from external regulation to a model that supports and provides feedback. SR can be important in the processes of healthy eating, as individuals who self-regulate their behavior are likely to plan and systematically use a set of cognitive and metacognitive strategies to meet self-set goals [29]. Thus, the effective use of SR strategies to achieve one’s goal is part of a key process in maintaining an individual’s motivation to reach a self-set goal, such as eating healthily [30].

In recent years, SR has started to receive attention as a key predictor of a variety health-related and wellbeing outcomes. For instance, poor SR appears to be a predictor of weight gain, especially in adolescence [18, 20, 31]. Several studies have successfully implemented self-management programs among individuals with various health conditions, such as heart disease [20]. Individuals enrolled in these programs were able to manage their health conditions and improve their quality of life and wellbeing. More recently, research showed that intervention programs designed to improve SR skills are among the most successful to attain healthy eating behaviors and may play an important role for long-term maintenance of this specific behavior [32]. For example, a study showed that a 2-year intervention combining information with SR strategies had a long-term effect on the consumption of fruits and vegetables when compared to the information-only intervention [33].

Nevertheless, evidence in this domain is still scarce. The majority of these programs focus on health promotion within a disease management context, not on prevention and promotion of a long-term healthy lifestyle, and target adults. Thus, the current proposal will address these gaps by focusing on healthy eating promotion, with a preventive emphasis, among elementary school-aged children.

### HEP-S

#### The intervention

HEP-S is a 20-week narrative-based intervention designed to promote healthy eating through the teaching and development of a set of transversal skills and strategies within that domain. Therefore, the intervention will not focus exclusively on conveying knowledge about healthy eating; the goal is rather to stimulate the agent role of children and promote their use of SR strategies and skills. The program will be implemented online using Canvas®. Canvas® is an online learning management system that allows to, among other things, create modules of content, attach files, share materials, create discussion forums, and conduct videoconferences. Each child will be provided with a unique login to the Canvas® of the group.

At the core of this intervention are the narratives. Narratives, or story-tools, are important educational tools that favor child development [34, 35]. They allow readers to reflect on themselves and their behavior through the characters; they instigate the debate and the uncovering of different perspectives on how to cope with everyday dilemmas [25]. Through the characters and plots, these story-tools create opportunities from which the readers can experience and develop autonomous behaviors and promote SR in a given domain (e.g., learning, healthy eating). Extensive extant research has examined and demonstrated the efficacy of using story-tools to promote SR strategies (e.g., [25, 29, 34]). In this study, two narratives will be used as the prompt for, and instigation of, discussion and reflection about healthy eating: specifically, *Yellow’s Trials and Tribulations,* by Rosário, Núñez, and González-Pienda [35], and *The Hill of the Bald Trees and Other Stories* (*O monte das árvores carecas e outras histórias)*, by Rosário, Magalhães, Pérez, and Arias [36].

The program has two main components: (i) the weekly synchronous session and (ii) the weekly parental involvement activity. Table 1 illustrates the anatomy of the intervention; it provides examples of the activities carried on during a typical week of the program, a brief description of that activity, and the rationale behind it.

**Table 1:** Anatomy of the online intervention, example of session activities and their rationale

W		Activity	Description	Rationale
**4**	SS	Reading and discussion of Chapter 1 - *YTT*	The chapter introduces the colors of the rainbow (the main characters of the story) and their psychological characteristics (e.g., Violet is strong and courageous).	Introduce the story’s characters and establish a parallel between the characters’ psychological characteristics and reflect how these characters may behave regarding their nutrition.
	PIA	What *color* is my family’s nutrition?	Children will be asked to choose between drawing the rainbow and matching the family members with the colors in terms of their nutrition; or writing about the association between the colors and their family members and their nutrition. In the end, children must take a photo and share it in the forum.	Get to know and become owner of the characters of the story to promote an identification with the plot.
**11**	SS	Reading and discussion of Chapters 9 and 10 – *YTT*	The pic-nic of the problems.	Behavior and emotions analysis; the importance of responsible acting and impulsivity management to solve non-adaptive strategies and behavior barriers and improve adaptive solutions.
	PIA	Barriers to healthy eating	Children will be asked to discuss in family and comment a scenario related to different problems that children face (e.g., lie, fear, tantrum, annoyance), such as “John makes a tantrum in almost every meal because he does not want to eat the soup”. Then, they have to express on the forum their thoughts on why this might happen, what are the consequences of such behavior, and suggest alternative behaviors.	Emotional and behavioral problem characterization, anticipation of consequences, and reflect on adaptive behavior alternatives.

Notes: *W* week, *FTF* face-to-face, *SS* synchronous session, *PIA* parental involvement activity, *YTT* Yellow’s Trials and Tribulations

##### The weekly synchronous session

For each intervention group of children, there will be every week a 60- to 75-min synchronous videoconference session managed by an educational psychologist with the role of facilitator. Each session will take place at each participants’ home, before or after school classes, during an hour selected by the participants. Parents can attend the session if they wish to do so, but cannot participate, and will be invited to partake in the weekly parental involvement task. Each synchronous session will be organized in three stages: (1) summarize last week’s chapter contents (e.g., the colors taught me what a plan is and how to do a plan) and revise previous week’s parental involvement activity, (2) read and explore the narrative in group through the facilitator’s questioning, and (3) reflect and formulate the session’s take-home message. Chapters of the *Yellow’s Trials and Tribulations* [35] and of *The Hill of the Bald Trees and Other Stories* [36] will be used in each synchronous session to prompt children’s metacognitive reflection about their own behavior through the characters’ behaviors. This strategy will allow children to discuss SR strategies in the healthy eating domain. The premise underlying this approach is that after participants discuss and reflect on the subject, they will undergo a change in their beliefs and comprehension of the “what,” “how,” “when,” and “why” of the phenomenon and, consequently, a behavior change [35]. In sum, we aim for children to become the main characters of their own stories, thus adopting an agent role in their health habits.

##### The weekly parental involvement activity

On a weekly basis, children will be prompted to deepen the learning on the SR skills and strategies for healthy eating that was worked through the narrative chapter in the synchronous session. This will be accomplished by instigating participants to complete activities on Canvas® together with their parents and family (e.g., categorize your pantry’s and fridge’s items according to the colors of the traffic light, take a photo, and share it in the forum with your colleague). It is expected that this activity boosts parents’ curiosity, contributes to parents’ involvement in the program, helps parents acquire the knowledge and strategies, and helps parents apply these strategies at home. The content will be released in the platform, and a facilitator (an educational psychologist with training on self-regulated learning (SRL)) will oversee interactions, provide individualized feedback, and promote children’s engagement in the program. Research shows that beliefs about healthy eating have a stronger impact on eating behavior than declarative knowledge about food [17]. The current project will address this concern by focusing on fostering SR skills and strategies about healthy eating. Still, general knowledge about food is necessary in order to know when to apply the SR strategies. Thus, the parental involvement activity will contribute to the promotion of a deep acquisition of declarative knowledge; i.e., factual knowledge will not be delivered directly to the child, it will be sustained by procedural knowledge about the topic [29, 37, 38].

#### The design

This is a 3-arm randomized controlled trial (RCT) with the following: a control group, with no program; an intervention group, with the online narrative-based program; and an enhanced-intervention group, with the online narrative-based program with gamification strategies embedded in the program. Both intervention groups (i.e., the intervention group and the enhanced-intervention group) will have the same narrative-based intervention described in Table 1. The only difference between the two intervention groups is that, in the enhanced online intervention group, gamification strategies will be added to the dynamics of the program. No other intervention components, timings, and materials will differ between these two trial arms. Despite the potential advantages of conducting online interventions, literature on digital interventions to promote behavior change has highlighted that this mode of delivery is associated with high rates of dropout [39]. Thus, one concern among researchers in this field is to devise strategies that mitigate the high rates of dropout and instill engagement in the online intervention [39, 40]. Accordingly, we are implementing the additional gamification strategies in the third group to assess the efficacy of these strategies in promoting engagement with the program and activities.

Gamification consists in the use of game design elements in a non-game context [41]. Non-game contexts can be education [42], health [43], and business [44]. Examples of game design elements are the narrative context, feedback, reputation, ranks, levels, competition, and relational support [41]. Considering the engaging nature of games, it is expected that adding some of their design and motivational features to the non-game contexts [41, 45, 46] will contribute to the (i) creation of engaging and fun contexts or environments, (ii) enhancement of the degree and depth of participants’ engagement, (iii) promotion of learning opportunities, and (iv) motivation of individuals to engage in the task at hand. Table 2 illustrates how gamification was infused in the online program for the enhanced online intervention group.

**Table 2:** Gamification elements used in the enhancement-treatment group

Elements*	Objective/rationale**	How it was implemented
Narrative context	Narratives contribute to user engagement. The narrative provides information about the characters and instigates reflection, as well as the establishment of a parallel between the characters’ actions and their own. It guides the behavior and organizes and provides meaning to the activities.	The narrative context within the intervention is created by the activities being conceived as an extension of the narrative/story-tool that children read during the program.
Feedback	Feedback contributes to user engagement. Feedback allows the user to know how things are going and provides hints on what the user needs to address in order to reach their self-set goals.	The educational psychologist provides personalized feedback to each interaction that children engage in their online group. Examples of feedback include: comments, instigation to reflect about some aspect of their behavior, praise of good choices, discourage not so good choices.
Reputations, ranks, and levels	Reputations, ranks, and levels contribute to user engagement. These elements show the users their place in the hierarchy of the group, promoting competition. It also informs other users about particular competencies or talents and sustained achievements that a user might have.	Children can earn points for performing each activity suggested in the platform. Every week there will be a ranking with the children that acquired points that week and the corresponding badge, as well as with information regarding the cumulative rank of the class. By accumulating points, children will progress and become closer to the end-goal.
Competition under rules that are explicit and enforced	Rules contribute to a sense of fairness among users. Rules allow competition to work when they are evenly and impersonally applied.	The rules are made explicit to children in the first session and available in the platform. The educational psychologist will oversee compliance with the rules and is the sole responsible for attributing points and badges, and making the ranking.
Teams	Teams contribute to user engagement. Teams allow interaction opportunities between members, who reveal their personalities and disclose personal experiences while collaborating to reach team goals.	Children are organized into small groups composed by classmates. Children earn points not only for performing the activities but also if their team members perform all the activities. By collaborating to reach team goals, each child will benefit with extra points in the cumulative rank
Time pressure	Time pressure contributes to users’ competition. Time pressure is one key element to create the sense of “uncertain winning conditions”.	At the end of each week the ranking will be made available with the results of each child’s activity for that week. Children will not be able to go back and complete activities that have already expired.

* These descriptors were retrieved from Deterding and colleagues [41]

** The description of the descriptors was based on Byron Reeves and J. Leighton Read article summarizing the “Ten Ingredients of Great Games”. http://www.cedma-europe.org/newsletter%20articles/misc/Ten%20Ingredients%20of%20Great%20Games%20(Apr%2010).pdf

### Goals and expected outcomes

The main purpose of this study is to evaluate the efficacy of an online intervention, narrative-based with or without gamification strategies, designed to promote healthy eating by fostering the use of SR strategies among elementary school-aged children. Thus, the major goals are to promote children’s use of SR strategies for healthy eating, to strengthen their sense of self-efficacy regarding healthy eating, and to increase the consumption of healthy foods. Other goals of the study include the following: increase knowledge about healthy eating and consequences of unhealthy nutrition or lifestyle; contribute to healthier food preferences; and contribute to more positive attitudes and perceptions about healthy eating. Thus, we want to assess the feasibility of conducting an online intervention with this population and examine the advantages of embedding the program with gamification strategies, by comparing it to the program without these strategies.

Overall, it is expected that children taking part of the intervention will, at the end of the intervention, increase their use of SR strategies for healthy eating, enhance their sense of self-efficacy for healthy eating, and increase their consumption of healthy foods. Additionally, it is expected that their knowledge on the topic will increase, that their food preferences become healthier, and that they will display perceptions and attitudes about the topic that are more positive. Lastly, it is expected that participants enrolled in the enhanced-online-intervention group will be more engaged in the intervention than the online-intervention group.

## Methods/design

### Design and procedures

The present protocol design followed the recommended procedures and items to address in a trial protocol, namely SPIRIT (Standard Protocol Items: Recommendations for Interventional Trials) 2013 checklist. As aforementioned, this is an RCT (REGISTRATION NUMBER: NCT04099498) with three groups: a control group, not enrolled in any program; an intervention group, enrolled in the online program; and an enhanced-intervention group, enrolled in the online program with gamification strategies. The sample will be recruited by first contacting public elementary and middle schools from Portugal and inviting them to participate. Orientation sessions will be held at schools for all parents of children in the target grades to explain the project and its rationale and to invite them to enroll their child. The study will be introduced as a program to promote healthy lifestyles among late elementary school-age children, which aims to monitor relevant engagement variables and examine children’s, parents’, and teachers’ perceptions of the utility and feasibility of these interventions.

### Retention and incentives for the participants

The following strategies will be undertaken to minimize attrition. First, prior to the enrollment of children in the intervention, the researchers will organize orientation sessions with parents to present the project and explain its pertinence and rationale. Second, parents who agree to enroll their child in the program will sign a participation agreement form. Third, parents will also be registered into the platform; moreover, reminder e-mails will be sent whenever there is an activity or task available in the platform, as well as the summary of the week’s chapter. Fourth, check-up sessions with parents will be carried mid-through the program. Lastly, to promote completion of follow-up phases, workshops will be offered to participant children, caregivers, and respective teachers about different themes (e.g., homework, inclusion of minorities). Nevertheless, participation is voluntary and, therefore, parents and children will be informed that they can withdraw from the program at any time. For that, participants only have to report their willingness to drop out of the program.

### Informed consent and randomization

During the orientation session, parents who allow their child to participate in the program will complete the first assessment protocol in loco, as well as complete the informed consent form. In the informed consent, the following aspects will be covered: (i) description of the program, accompanied by a timeline, (ii) duties of the parents as guarantors of their child’s involvement in the program, and (iii) what is expected from parents regarding their involvement with the program and its activities. Children will also complete an informed consent form. Additionally, participants will be asked if they agree to the use of their data in case they choose to withdraw from the trial. Participants will be asked for permission for the research team to share relevant data with people from the Universities taking part in the research or from regulatory authorities, where relevant (e.g., the dissemination plan will be presented).

Genuine randomization at the individual level will not be possible as participants originating from the same class and school will have to be allocated to the same treatment condition to prevent between-group contamination. To address this difficulty, each school that accepts to enroll in the study will be randomly attributed a number associated to one of the groups (i.e., control, intervention, and enhanced-intervention) in a 1:1:1 basis. Moreover, for the intervention groups, small groups of five participants each will be randomly created though computed-generated random numbers.

### Participants

Children from the 5th and 6th grades will be recruited. Children, instead of adolescents, were selected because it is during childhood that the formation of habits and behaviors takes place [47]. Moreover, unlike children, adolescents are on a developmental stage where a sense of autonomy arises, and many adolescents are resistant to interventions [48, 49]. That is, among adolescents, external control (e.g., parents) of food consumption decreases and internal control becomes increasingly predominant in the face of food choices. Moreover, considering the required degree of autonomy and skills on ICT to partake in the study, the late childhood spectrum seemed more feasible to implement the program.

For the purpose of this study, children attending the regular curriculum will be included. We will seek to exclude children attending alternative curricula, i.e., who have been identified by the school office as having special needs, since these children will not have the necessary autonomy required to attend the program. Additionally, despite the preventive nature of the present program, children will not be included/excluded from the intervention based on their weight status, nor will they need an alteration of their usual care pathways (e.g., medication) to participate in the intervention. Specific inclusion criteria include the following:

1. Access to a computer equipped with a camera and speakers at home;
2. Internet access at home;
3. Parents have to provide a written consent for their children to participate;
4. Children have to provide a written agreement of willingness to participate;
5. Parents have to be willing to participate in parental involvement activities;
6. Parents have to own an e-mail account or be willing to create one.

### Proposed sample size

Considering this longitudinal design, in the calculation of the sample, we considered a 0.25 effect size, an alpha level of 0.05, a desired statistical power of 0.8, five measures, and three research groups (i.e., control, online intervention, enhanced online intervention). To run a three-level cluster-randomized trial, previous analysis suggested 40 groups per group condition with 15 participants in each, measured at five different time points (see section “Definition of the hierarchical model”).

### Assessment

Table 3 illustrates the timeline for the different assessment measures of the children participating in this project.

**Table 3:** Timeline for the different assessment measures of the project

Measures	Registration (−T1)	Baseline assessment (T0)	Final assessment (T1)	3-month follow-up assessment (T3)	6-month follow-up assessment (T4)	Weekly assessment
Eligibility screen	X	–	–	–	–	–
Informed consent	X	–	–	–	–	–
Anthropometric	X	–	X	X	X	–
Sociodemographic	X	–	X	X	X	–
Self-Regulation^1^	–	X	X	X	X	–
Self-efficacy^2^	–	X	X	X	X	–
Declarative knowledge^3^	–	X	X	X	X	–
Attitudes and perceptions^4^	–	X	X	X	X	–
Healthy eating and physical activity behaviors^5^	–	X	X	X	X	–
Satisfaction	–	–	Intervention groups	–	–	–
Journal	–	Control group	Control group	–	–	Intervention groups

*X* available to all groups

^1^Self-Regulation Processes towards Healthy Eating Questionnaire

^2^ Self-Efficacy to Regulate Eating Habits for Children Questionnaire & Healthy Eating and Physical Activity Self-Efficacy questionnaire for children

^3^ Knowledge of Healthy Eating Questionnaire

^4^Students’ Attitudes and Perceptions on Healthy Eating Questionnaire

^5^ Healthy Eating and Physical Activity Behavior Recall Questionnaire for Children

### Anthropometric measures

Children participating in the intervention and control groups will have their weight, height, and waist circumference measured by the research team at the baseline, post-intervention, and at the 3- and 6-month follow-ups. This trial does not involve collecting biological specimens for storage.

### Self-report questionnaires

#### Sociodemographic measures

These measures will include questions about participants’ sex, age, grade, academic achievement, and socioeconomic level, as well as questions about other healthy habits and behaviors, such as their daily screen time and physical activity.

#### Psychological self-report measures

##### Self-Regulation Processes towards Healthy Eating Questionnaire

An adapted version of *Self-Regulation for Health* scale [50] will be used to assess the SR processes towards healthy eating. This measure consists of nine statements regarding the participants’ self-regulation towards healthy eating (e.g., “I plan my meals. I think about what I’m going to eat and what it takes to prepare my meal”). Responses to the individual items are scored from 1 (never) to 5 (always) in a Likert-like scale and summed to create a composite score ranging from 9 to 45, with higher scores implying more self-regulation.

##### Healthy Eating and Physical Activity Self-Efficacy questionnaire for children (HEPASEQ-C)

An adapted version of the HEPASEQ-C will be used to assess self-efficacy related to enacting healthy eating and activity behaviors in children [51]. This version consists of nine items, with seven focusing on self-efficacy related to healthy eating (e.g., “I will eat healthy food even when my friends eat food that is not healthy.”), and two focusing on self-efficacy related to physical activity (e.g., “I am physically active for 60minutes/ 1 hour per day”). Responses to the individual items are on a Likert-like scale, ranging from 1 (There is no way I can do this) to 5 (I am definitely sure that I can do this) and summed to create a composite score ranging from 9 to 45, with higher scores implying more self-efficacy. Also, a sixth option was added when the situation is not applicable. This answer does not contribute to the composite score.

##### Healthy Eating and Physical Activity Behavior Recall questionnaire for Children

This measure was developed as a complement to the HEPASEQ-C questionnaire and aimed to recall children’s behaviors in terms of healthy eating and physical activity [51]. The adapted version of this questionnaire consists of 19 items. Five items are in an open response format allowing children to write the actual foods they ate (e.g., “Last time I had a meal at a friend’s place I ate…”). Six items are yes/no response options (e.g., “I eat breakfast every day”). For the remaining eight items, responses were provided via writing down a number (e.g., “Over the last 3 days I drank … soda beverages”). Frequencies of responses to the individual items will be analyzed (e.g., percentage of students that eat breakfast, percentage of students that choose healthy options at friends’ home).

##### Self-Efficacy to Regulate Eating Habits for Children

An adapted version of the questionnaire “Self-Efficacy to Regulate Eating Habits” developed by Bandura [52] will be used to assess student’s perceived capability to regulate and to adopt healthy eating habits in different daily situations (e.g., “While watching television”). This version consists of 17 adapted situations/scenarios, plus the option of providing other examples, in which participants will have to respond if they can choose healthy food choices or not. Responses to the individual items are presented in a Likert-like format, scored from 1 (not capable) to 5 (capable, for sure) and are summed to create a composite score ranging from 17 to 85, with higher scores implying more self-efficacy to regulate eating habits. Also, a sixth option was added when the situation is not applicable. This answer does not contribute to the composite score.

##### Attitudes and Perceptions on Healthy Eating Questionnaire

An adapted version of the Students’ Attitudes and Perceptions on Health Instrument [50] will be used to assess students’ attitudes and perceptions towards healthy eating. This measure consists of 17 statements about student’s attitudes and perceptions of the importance of healthy eating (e.g., “Eating fruit and vegetables will help me growing up”). Responses to the individual items are presented in a Likert-like format, scored from 1 (I completely disagree) to 5 (I completely agree) and are summed to create a composite score ranging from 17 to 85, with higher scores implying more positive attitudes and perceptions about healthy eating. Also, a sixth option was added when the situation is not applicable. This answer does not contribute to the composite score.

##### Knowledge for Healthy Eating Questionnaire [37]

This questionnaire was developed to assess school-age children’s (6 to 17 years old) declarative knowledge about healthy eating. The questionnaire consists of 15 statements and participants have to rate their agreement regarding each statement (e.g., “Our meal should contain varied and colourful foods”; “Going to school without having breakfast does not interfere with my school performance”). Responses to individual items are scored from 1 (totally disagree) to 5 (totally agree) in a Likert-like format. Responses of each participant are summed to create a composite score ranging from 15 to 75, with higher scores implying more knowledge of healthy eating. The alpha of Cronbach of the original study was 0.73.

##### Satisfaction questionnaire

The research team will develop a satisfaction questionnaire based on previous satisfaction instruments used with this population. The questionnaire will address aspects of utility and feasibility of the program and perceived support by the educational psychologist from the students’ perspective.

#### Weekly assessment

##### Journal

On a weekly basis, children will complete an online journal with specific questions regarding the program and their behaviors. The weekly journal will allow monitoring the individual progress of each child in the program and, simultaneously, to create another moment to reflect about, and consolidate, the learning acquired in the program. Questions will be divided in three sections: (a) invite children to reflect on what they have learnt with the narrative, the synchronous session, and the parental involvement activity of that week according to three types of knowledge [declarative (What I have learnt); procedural (How can I use what I have learnt); and conditional (When can I use in my daily routines what I have learnt)]; (b) questions regarding participants’ cognitive, emotional, and behavioral engagement in the program during the week will also be included; and (c) concrete questions about their food intake will also be covered in the weekly journal.

##### Engagement

Engagement in the program will be assessed with observational measures though the analysis of the interactions of children in the online platform [53]. Examples include number of activities completed, comments to peers’ posts, and others.

### Primary and secondary outcome measures

The primary outcomes will be the analysis of differences in SR, self-efficacy, knowledge towards healthy eating, and, additionally, the amount of healthy and unhealthy food consumption at baseline (*T*_0_), final (*T*_1_), and follow-up (*T*_3_, *T*_4_) assessments. Secondary outcome measures will include the anthropometric (i.e., body mass index [BMI] *Z*-scores), sociodemographic variables (−T_1_), other healthy behaviors (e.g., screen time and physical activity), psychological (e.g., attitudes and perceptions about healthy eating), and engagement (e.g., weekly journal) measures. Differences within the group (i.e., at baseline, final, and follow-ups assessments), as well as between groups (i.e., control, online-intervention, and enhanced-online-intervention), will be tested over time. The repeated measures assessment allows testing relevant factors affecting SR and self-efficacy on healthy eating and food consumption. Additionally, weekly engagement indicators collected with the journals and with the actual interactions with the platform will be tested as predictors of primary outcome measures in final and follow-up assessments. Lastly, moderators and mediators that can affect the outcomes of the intervention but that are not predictor variables include sex, socioeconomic level, and academic achievement.

### Statistical analysis plan

The effectiveness of the HEP-S intervention will be assessed using linear mixed-effect models. Specifically, we will assess the impact of interventions groups vs. control group on changes over time regarding the primary outcomes (e.g., healthy eating behavior, SR, self-efficacy towards healthy eating). For that, we will first examine within-intervention group differences (i.e., over time) followed by between-intervention group assessments at each time point. Differences between groups will be supported if the beta-weight parameter for the interaction between group and time are statistically significant.

Moreover, statistics descriptive analyses will be computed to describe all participants’ characteristics by using chi-square tests and independent tests to compare groups on the distributions according to sex, age group, BMI *Z*-score classification, and others. In addition, secondary outcomes will be analyzed by using similar models, comparing the three research groups. Also, these secondary repeated measures (e.g., satisfaction with the program) will be examined by conducting an analysis of covariance to explore interference with main outcomes. Finally, no interim analyses of the primary and secondary outcomes are planned to be included in this trial.

All analyses will be completed by using SPSS software (version 26), and *p* values below .05 will be considered statistically significant. The normality, homoscedasticity, and linearity of the residuals of each model will be examined to ensure that the assumptions of the models are met.

### Definition of the hierarchical model

To evaluate the effectiveness of program, a three-level cluster-randomized trial is considered where classrooms are randomly assigned to treatment and control groups. Let *Y*_*tij*_ denote the response at time *t* for *i*th participant (*i* = 1,…, *n*_*j*_) in the *j*th group (*j* = 1, …, *J*), e.g., the *i*th student in the *j*th classroom. The three-level model for longitudinal experiments that involve clustered data is as follows:

1 $$ \mathrm{Level}-1:\kern0.5em {Y}_{tij}={\uppi}_{0 ij}+{\uppi}_{1 ij}{T}_{tij}+{e}_{tij},\kern0.5em {e}_{tij}\sim N\left(0,{\sigma}_{et}^2\right) $$

2 $$ \mathrm{Level}-2:\kern0.5em {\displaystyle \begin{array}{l}{\pi}_{0ij}={\beta}_{00j}+{r}_{0 ij}\\ {}{\pi}_{1ij}={\beta}_{10j}+{r}_{1 ij},\end{array}} $$

3 $$ {\displaystyle \begin{array}{l}\mathrm{Level}-3:\kern0.5em \begin{array}{l}{\upbeta}_{00j}={\gamma}_{000}+{\gamma}_{001}{G}_j+{u}_{00j}\\ {}{\upbeta}_{10j}={\gamma}_{100}+{\gamma}_{101}{G}_j+{u}_{10j},\end{array}\\ {}\left[\begin{array}{l}{r}_{0 ij}\\ {}{r}_{1 ij}\end{array}\right]\sim N\left[\begin{array}{l}0\\ {}0\end{array}\right],\left[\begin{array}{ll}{\sigma}_{r0}^2& \\ {}0& {\sigma}_{r1}^2\end{array}\right],\left[\begin{array}{l}{u}_{00j}\\ {}{u}_{10j}\end{array}\right]\sim N\left[\begin{array}{l}0\\ {}0\end{array}\right],\left[\begin{array}{ll}{\sigma}_{u0}^2& \\ {}0& {\sigma}_{u1}^2\end{array}\right]\end{array}} $$

Substituting Eqs. 2 and 3 into Eq. 1, we have the mixed model of interest

4 $$ {Y}_{tij}={\gamma}_{000}+{\gamma}_{00j}{G}_j+{u}_{00j}+{r}_{0 ij}+{\gamma}_{100}{T}_{tij}+{\gamma}_{101}{G}_j{T}_{tij}+{u}_{10j}{T}_{tij}+{r}_{1 ij}+{e}_{tij} $$

The lowest level variance component is represented by *e*_*tij*_, and the two level 2 variances are as follows: the variability in intercepts across subjects (*r*_*0ij*_) and the variability in slopes across students (*r*_*1ij*_) nested within classrooms. The level 3 variance components are as follows: the variability in intercepts across classrooms (*u*_*00j*_) and the variability in slopes across classrooms (*u*_*10j*_).

As Heo et al. [54] and others (e.g., [55]) have indicated, the most important goal of a longitudinal intervention is to test whether there are differences between intervention groups with respect to their average growth rates. Applying the approach of these authors, the power to detect a specified treatment difference between average rates of change for the groups is defined as the probability of rejecting the null hypothesis of no treatment-by-linear-trend interaction *H*_0_: γ_101_ = 0, given that it is fact false (γ_101_ ≠ 0). This hypothesis can be tested with

5 $$ {F}_o=\frac{{\hat{\gamma}}_{101}^2}{Var\left({\gamma}_{101}\right)}=\frac{\Delta^2\left[{N}_1{N}_2{N}_3 Var(T)\right]}{2\left(1-{\rho}_1\right){\sigma}^2{N}_1 Var(T){\sigma}_{u1}^2}, $$

where Δ is the effect standardized effect size at the last time point, *N*_1_ is the number of level 1 units (repeated measures) per student, *N*_2_ the number of level 2 units (students) per classroom, *N*_3_ the number of level 3 units (classroom) per treatment condition, *Var*(*T*) is the population variance of the time variable *T*, ${\upsigma}_{u1}^2$ is the three-level random slope variance, σ^2^ is the sum of the other variances, and *ρ*_1_ is the correlation between outcomes measures at different time points on the same student nested within classroom.

The power of the test statistic *F*_0_, denotes 1 − *β*, can be written as follows:

6 $$ 1=\beta =\phi \left\{{\left\{\frac{\Delta }{\left({N}_1-1\right)}\left[\frac{\left[{N}_1{N}_2{N}_3 Var(T)\right]}{2\left(1-{\rho}_1\right){\sigma}^2{N}_1 Var(T){\tau}_s}\right]\right.}^{\frac{1}{2}}-{Z}_{1-\left(\alpha /2\right)}\right\}, $$

where τ_*s*_ is the ratio of the three-level random slope variance to the sum of the other variances, ϕ is the cumulative distribution function of a standard normal distribution, and *Z*_1 − (α/2)_ is the 100 (1 − *α*/2) percentile of the standard normal distribution for a bilateral test.

Consider the following hypothetical values for three-level cluster-randomized trial design parameters: ${\upsigma}_e^2=0.5$, ${\upsigma}_{r0}^2=0.3$, ${\upsigma}_{r1}^2=0.0$, ${\upsigma}_{u0}^2=0.2$, and ${\upsigma}_{u1}^2=0.1$. Under the fixed slopes model, the correlation between the outcomes from different student nested within the same classroom is ${\uprho}_2={\upsigma}_{u0}^2/\left({\upsigma}_e^2+{\upsigma}_{r0}^2+{\upsigma}_{u0}^2\right)=0.2$, whereas the correlation between the outcomes measured at different time points on the same students nested within classrooms is ${\uprho}_1={\upsigma}_{r0}^2+{\upsigma}_{u0}^2/\left({\upsigma}_e^2+{\upsigma}_{r0}^2+{\upsigma}_{u0}^2\right)=0.5$. Further assumptions are as follows: (a) number of classrooms per treatment condition: *N*_3_ = 40; (b) average number of students per classroom: *N*_3_ = 15; (c) number of repeated measures per student: *N*_1_ = 5; (d) alpha significance level: *α* = 0.05; and (e) statistical power: 1 − *β* = 0.80.

Given design parameters and further assumptions, the minimum detectable effect size is 0.25. In our opinion, an effect size of Δ = .25 is a good first estimate of the smallest effect size of interest in psychological research, since it is not irrelevant and requires a considerable sample size to detect a treatment effect predicted by the researcher.

Finally, we consider that a study with 80% power is a properly powered study, despite several researchers (see, e.g., [56]) consider this a value rather low, as it entails a 20% chance of not finding a theoretically important finding.

### Blinding and data access

The assessment protocol will be completed by children on-site and will not be available for parents to consult or to the educational psychologist conducting the sessions. Each child will be attributed a unique code. Moreover, data will be analyzed by an external statistician who will be blind to the research groups of each data set. Additionally, only the principal investigator and the statistician will have access to the full data set, which will be used only for investigation. Also, all questionnaires will be destroyed once all data are published. Lastly, the procedures for personal data storage, handling, and protection will comply with the newest GDPR (General Data Protection Regulation) policies. Particularly, to ensure privacy and anonymity, pseudonymization will be carried out. This is a process that transforms personal data into a data set that cannot be linked to a particular subject unless another piece of information is added (e.g., decryption key). Usually, these data are stored in different locations.

### Data monitoring and trial management

The trial will be supervised by a Trial Steering Committee (TSC) directed by the principal investigator (PI) of the trial. The TSC will meet every 4 months to monitor the trial progress, as well as review and manage the data. Besides the PI, the TSC will also include two independent researchers, one educational psychologist and a methodology expert, who are not directly involved in the study, and the CoPI. An independent statistician with no involvement in the trial will conduct all the data analysis and will inform the results to the TSC at a joint meeting. The co-investigators and research assistants of the project will be responsible for all aspects of local organization of the trial (e.g., identify potential recruits, take consent). To ensure the protocol is implemented as planned, the PI will be responsible for managing and supervising the trial and providing direction and administrative support. Three general meetings with the entire group of researchers will be held throughout the project and smaller monthly meetings will be conducted between the PI, the CoPI, and the researchers to monitor the development of activities and ensure compliance with the goals. Lastly, regular meetings will be conducted between the PI, the science manager, and the financial manager of the research center.

This trial does not have a Data Monitoring Committee (DMC) as the current trial is a low-risk intervention, without safety issues or foreseen risks associated. Moreover, the trial does not have a Stakeholder and Public Involvement Group (SPIG) as the researchers that will carry the intervention are trained and certified educational psychologists.

### Safety aspects and ethical considerations

The standard procedure to conduct studies and interventions in the Portuguese school context requires an a priori evaluation and validation of the project by the Ministry of Education before researchers can invite schools to participate. Additionally, approval of the Ethics Committee of the University of Minho was obtained and can be consulted on the trial registration. Participation in the program is entirely voluntary, and parents and children are informed that their involvement in the school’s activities will not be affected by their decision on whether or not to participate. Additionally, because there are no anticipated detrimental consequences or real risks for the participants involved in this intervention, we did not include in the current trial interim assessment, stopping rules, nor criteria for discontinuing or modifying allocated interventions. In fact, parents and children are informed they can withdraw from the program at any time. We fully recognize our responsibilities for child protection, so the project must ensure that the children who participate are safe from all forms of abuse, injury, neglect, maltreatment, and exploitation. The project will ensure an environment where children feel secure, feel free to either participate or leave the study at any time, are encouraged to talk, and are listened to. Child welfare is paramount. Hence, children are safeguarded by the adoption of child protection guidelines and by a code of conduct for all involved. In order to work with children, researchers must present their criminal records during the job application. Additionally, parental consent must be obtained before carrying any procedure with children. This protocol will be held to meet high ethical standards.

Lastly, for ethical reasons, once all the assessments have been conducted, the control group will be given the opportunity to receive the intervention. This will guarantee that all children share the same benefits of the program. In this sense, all participants will have the opportunity to develop not only knowledge but also a set of transversal skills and strategies on the healthy habits’ domain. The participation in this intervention will bring no harm, and no risks were identified. Therefore, there will be no compensation for trial participation, and no post-trial care for participants is anticipated.

### Dissemination

The plan for disseminating the research outcomes of the HEP-S program include the following: (a) write and publish papers in peer-reviewed journals, including the efficacy of the intervention program; (b) write a document systematizing guidelines for online interventions focused on the promotion of healthy eating through SRL; (c) conduct workshops about implications for practice targeting participants (both children and caregivers), educators, health professionals, and relevant stakeholders; and (d) organize advocacy groups comprised of children, parents, teachers, and the health community to advocate for the importance of launching policies on healthy eating habits. Advocacy groups for healthy eating habits could consider working with governmental agencies and food companies to help set environmental and structural changes likely to facilitate individual healthy choices. The work done by these advocacy groups is expected to generate public debate on this issue and, hopefully, help flourish innovative strategies to prevent obesity.

## Discussion

Prevention of overweightness and obesity remains poorly effective, representing a great challenge and a major concern for governments and relevant stakeholders worldwide. Despite the numerous efforts carried to mitigate this major health issue, overweightness and obesity do not seem to be diminishing [1]. There is, therefore, a pressing need to provide evidence-based options to improve healthful eating among individuals of all ages, particularly among children.

This study will develop and evaluate a preventive online-based intervention to promote the development of self-regulation skills for healthy eating among elementary school children. Although many online preventive interventions regarding healthful lifestyles have been developed, seldom the approaches have focused on children or skill development. Moreover, although the program has children as the target population, parents and families will be deeply involved throughout the intervention. All these aspects confer to this intervention a unique and innovative character.

An online-based intervention will, expectedly, help overcome some challenges associated with the implementation of school-based programs. For instance, educational psychologists are still a scarce resource at the school setting, being often difficult to develop programs of preventive nature instead of remedial ones. Thus, it is aimed that HEP-S helps narrow the gap between children and their access to preventive, skill promotion, psychoeducational programs, in a cost-effective format by providing at-distance support to several groups of children. This strategy allows allocating human resources more effectively as, often, an education psychologist is responsible for several schools within the same district. Furthermore, it is expected that by actively involving parents in the intervention, adherence and engagement raises. Thus, we believe the HEP-S program may play an important role in preventing risky and unhealthy eating behaviors and contribute to more healthful lifestyles within families.

This study has some strengths worth highlighting. First, it presents an online-based intervention focused on promoting SR skills and strategies to promote healthy eating behaviors among elementary school-aged children. Second, it is conceived as a preventive approach, focused on promoting health-related behaviors, not on treatment. Third, gamification strategies will be implemented to reduce high rates of attrition and low feelings of engagement with the tool. Lastly, the research design foresees an implementation period longer than most of the intervention studies in this domain, with long follow-up. This will allow searching for sustained behavioral changes as a result of the intervention.

There are potential limitations to the present study proposal. First, although we include a weekly journal for participants to complete, during the study it will be difficult to monitor the actual implementation of the strategies trained in the program. Second, full adherence to the program will be a challenge as it implies some level of parental involvement and it lasts for 20 weeks. Third, although the study design is optimized by the selection of relevant psychological variables and the introduction of gamification strategies, the small sample size of this study confers a limited scope to examine full effects. In fact, there is the possibility that smaller effects than anticipated are found. This particular aspect may pose limitations when reflecting about the implications and conclusions of the study. Nevertheless, the study attempts to minimize this aspect by having conducted a priori power analyses. If the present trial shows positive effects at the primary outcomes level, future trials should try to use larger samples, from distinct backgrounds and cultures and include longer follow-up measures (> 6 months).

Altogether, we expect that the results of this study will provide evidence on whether training and fostering SR strategies among elementary school-aged children helps in the promotion of healthy eating habits.

## Trial status

The trial has been approved by the University of Minho Ethics Committee for Social and Human Sciences Research (CEICSH 032/2019) and then registered at the ClinicalTrials.gov (NCT04099498, https://clinicaltrials.gov/ct2/show/NCT04099498) on September 23, 2019. At the time of submission, this trial was not yet recruiting participants.

This is version 2.0 of the protocol. The date of submission: 7 July 2020.

The planned dates for recruitment are January 2020 and completion of recruitment July 2020.

## Data Availability

Trial results will be published in peer-reviewed journals and will be presented at national and international conferences. The complete datasets analyzed during the current study are available from the corresponding author on reasonable request.

## Abbreviations

HEP-S: Healthy Eating Program through Self-Regulation
ICT: Information and communication technologies
SR: Self-regulation
SRL: Self-regulation learning
RCT: Randomized controlled trial
BMI: Body mass index
GDPR: General Data Protection Regulation
WHO: World Health Organization
